# Flatland Metasurfaces for Optical Gas Sensing

**DOI:** 10.3390/s26041293

**Published:** 2026-02-17

**Authors:** Muhammad A. Butt

**Affiliations:** Institute of Microelectronics and Optoelectronics, Warsaw University of Technology, Koszykowa 75, 00-662 Warsaw, Poland; ali.butt@pw.edu.pl

**Keywords:** flatland, metasurfaces, gas sensing, visible, THz

## Abstract

Flatland metasurfaces provide a fundamentally distinct approach to optical gas sensing by confining light–matter interaction to planar, subwavelength interfaces, where resonant energy storage and near-field enhancement replace extended optical path lengths. This review presents a physics-driven perspective on metasurface-enabled gas sensing, focusing on how gaseous analytes perturb the complex eigenmodes of engineered planar resonators. Diverse sensing modalities, including enhanced molecular absorption, refractive index-induced resonance shifts, loss modulation, polarization conversion, and chemo-optical transduction, are unified within a common perturbative framework that links sensitivity to mode confinement, quality factor, and analyte overlap. The analysis highlights fundamental trade-offs imposed by material dispersion, intrinsic loss, and radiation balance across plasmonic, dielectric, polaritonic, and hybrid metasurface platforms operating from the visible to the terahertz regime. Attention is given to the limits of chemical selectivity in flatland architectures and to the role of functional materials, multimodal transduction, and computational inference in addressing these constraints. System-level considerations, including thermal stability, fabrication tolerance, and integration with detectors and electronics, are identified as critical determinants of real-world performance. By consolidating disparate approaches within a unified flatland framework, this review provides physical insight and design guidance for the development of compact, integrable, and application-specific optical gas sensing systems.

## 1. Introduction

The detection of gaseous species underpins a broad range of scientific and technological domains, including environmental monitoring, industrial process control, public safety, and healthcare [1,2,3]. Accurate gas sensing requires platforms that can provide sufficient sensitivity, selectivity, temporal response, and long-term stability while remaining compatible with increasingly stringent constraints on size, power consumption, and system complexity [4,5,6,7,8]. Conventional gas sensing approaches, such as bulk optical absorption cells [9], photoacoustic systems [10], and chemically sensitive electrical sensors [11], including chemiresistive [12], electrochemical [13], field-effect transistor (FET)-based catalytic combustion [14], and frequency-based sensors such as Quartz crystal microbalance (QCM) and surface acoustic wave (SAW) [15,16,17], have enabled reliable detection across many applications but typically rely on macroscopic interaction volumes or complex auxiliary instrumentation. These characteristics can limit miniaturization and dense integration in certain application contexts, posing challenges for dense spatial deployment, and seamless integration with modern electronic and photonic systems [18].

Recent advances in nanophotonics have introduced metasurfaces (MSs) as a powerful alternative platform for controlling light–matter interaction within ultrathin planar geometries [19,20,21]. MSs consist of arrays of subwavelength resonant elements whose collective optical response is defined primarily by geometry rather than bulk material properties [22]. By engineering resonance frequency and field confinement, MSs enable precise control of optical amplitude and phase. Additional control over radiation loss and symmetry further allows manipulation of polarization and spectral response [23,24,25,26]. These capabilities have motivated intense research into MS-based sensing, where small perturbations in the local electromagnetic environment can be converted into measurable optical signals [27]. Within this context, the concept of flatland optics has emerged as a unifying framework to describe photonic systems in which light–matter interaction is confined to planar, deeply subwavelength interfaces rather than extended propagation paths [28,29,30,31,32,33]. In flatland platforms, optical interaction strength is governed by resonant energy storage, near-field enhancement, and surface overlap with the analyte, instead of by physical path length [34,35]. For gas sensing, this paradigm is particularly compelling, as gaseous molecules typically interact weakly with light and therefore benefit disproportionately from enhanced local electromagnetic fields [36,37]. In contrast to biosensing platforms, where analytes are often immobilized near the sensing interface [38], gas sensing involves low-density, freely diffusing molecules, making near-field overlap, adsorption dynamics, and surface accessibility critical performance constraints. These characteristics place stronger emphasis on field confinement and analyte–mode overlap than in many conventional MS applications such as imaging, modulation, or wavefront control [39,40,41]. MSs naturally embody the flatland concept by localizing optical modes at engineered surfaces, where gas-induced perturbations are maximized [42,43].

**Figure 1:**
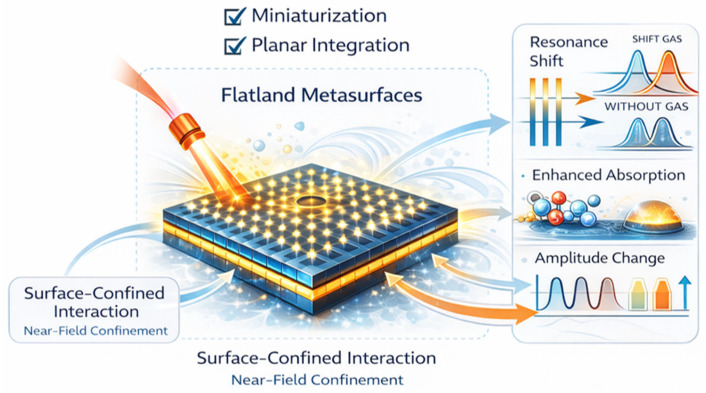
Conceptual framework of flatland MSs for optical gas sensing.

Gas sensing with MSs has rapidly diversified beyond a single physical mechanism [44,45]. Depending on design and material choice, MSs can transduce gas presence through enhanced molecular absorption, resonance frequency shifts driven by refractive index changes, absorption-induced damping and amplitude modulation, polarization conversion, wavefront reconstruction, or material-mediated chemo-optical effects [46]. In some implementations, MSs act directly as the interaction medium that enhances coupling between light and gas molecules, while in others they serve as optical transducers that amplify or visualize changes originating in functional layers such as liquid crystals, metal oxides, graphene, or porous frameworks [44,45,47]. This diversity of underlying mechanisms underscores MSs as a versatile and integrative platform, rather than a narrowly circumscribed sensing technology [48,49,50]. Despite rapid experimental advances, the field remains fragmented across spectral regimes, material platforms, and sensing paradigms, with limited cross-comparison and a lack of a shared physical framework [51,52]. As a result, it can be challenging to assess how different MS-based gas sensors relate to one another, what trade-offs govern their performance, and which approaches are best suited for specific applications. A comprehensive perspective that organizes MS gas sensing by underlying physical transduction mechanisms rather than by isolated device demonstrations is, therefore, timely and necessary. Figure 1 schematically illustrates the flatland MS paradigm for optical gas sensing that underpins this review. In practical implementations, this flatland interaction principle enables measurable gas detection by converting gas-induced permittivity perturbations into resonance shifts, absorption changes, or damping variations that scale with gas concentration.

This review presents a systematic, physics-driven overview of flatland MSs for optical gas sensing. Section 2 establishes the physical framework governing gas–MS interactions, highlighting the roles of electromagnetic field confinement, resonance quality factor, and analyte overlap. Section 3, Section 4 and Section 5 survey core transduction mechanisms, including resonantly enhanced molecular absorption and spectroscopy (Section 3), refractive-index-induced resonance-shift sensing (Section 4), and loss-damping or amplitude-modulation-based detection (Section 5). Material-mediated and chemo-optical flatland sensing strategies are discussed in Section 6, followed by polarization-, wavefront-, and visually encoded sensing concepts in Section 7. Section 8 compares MS operations across spectral regimes and material platforms, clarifying fundamental trade-offs between sensitivity, selectivity, and loss. MS architectures and design strategies are analyzed in Section 9, emphasizing plasmonic, dielectric, and hybrid platforms. Section 10 addresses system-level integration and application perspectives, and Section 11 concludes with remaining challenges and outlook. By unifying these diverse approaches within a common flatland framework, this review clarifies the role of MSs as optical transducers for gas sensing and guides the rational design of next-generation compact, integrable sensing systems.

## 2. Physical Framework of Gas MS Interaction

In flatland MS platforms, optical gas sensing arises from the sensitivity of planar resonant modes to perturbations introduced by nearby gaseous environments [51]. MSs support engineered electromagnetic eigenstates whose energy is spatially concentrated within subwavelength regions near the surface [53,54,55]. When gas molecules occupy these regions, they modify the local electromagnetic conditions experienced by the mode, resulting in detectable changes in the optical response [56,57,58]. The nature of this response depends on how the gas couples to the mode and which physical property of the resonance is most strongly affected [59].

A general description of this interaction can be formulated by considering how gas exposure alters the complex permittivity sampled by the resonant field [60,61]. Modifications to the real component primarily influence phase accumulation and resonance frequency, while changes to the imaginary component introduce additional dissipation and reduce photon lifetime [62]. These effects may originate from intrinsic molecular absorption, variations in gas density, adsorption at interfaces, infiltration into porous or polymer layers, or gas-driven changes in the optical properties of functional materials integrated with the MS [63]. Unlike biosensing platforms, where analyte localization is often externally controlled or surface-bound, gas sensing performance is strongly influenced by adsorption–desorption equilibrium and environmental variability, directly linking electromagnetic design to surface chemistry and operating conditions. Although the underlying microscopic mechanisms may vary, their macroscopic optical effect is invariably a modification of the resonance condition, allowing diverse sensing strategies to be interpreted within a unified physical framework.

The observable signal is determined by how this perturbation reshapes the balance between energy storage and loss within the resonant system. In modes with strong radiative coupling, small environmental changes can significantly alter interference conditions and produce pronounced spectral or intensity modulation [64]. In contrast, modes dominated by intrinsic dissipation respond less efficiently to additional gas-induced loss, limiting achievable sensitivity [65]. This distinction is particularly relevant when comparing plasmonic MSs, where ohmic loss sets a broad linewidth floor, with dielectric or polaritonic platforms that support longer-lived resonances but typically exhibit weaker absolute field enhancement [66,67]. Hybrid designs redistribute electromagnetic energy between low-loss and strongly confining regions to navigate this balance [68,69].

From a design perspective, sensing performance is governed by three coupled parameters: resonance quality factor [20,27], electromagnetic mode volume, and overlap between the modal field and the region affected by the gas. High-quality factor resonances improve the resolvability of small perturbations, while reduced mode volume increases interaction strength per unit analyte volume [20,55]. The overlap factor determines how efficiently the gas perturbs the stored energy, which is especially critical in flatland systems where interaction occurs within a thin near-surface region rather than along an extended optical path. Enhancing these parameters simultaneously is challenging, as strong confinement and narrow linewidths increase sensitivity to fabrication imperfections, temperature variations, and spectral misalignment [70].

Distinct sensing modalities emerge by emphasizing different elements of this parameter space. Absorption-based approaches maximize coupling to molecular loss channels and are most effective when optical resonances coincide with intrinsic gas fingerprints [42]. Resonance shift sensing treats the gas as a refractive perturbation and tracks changes in eigenfrequency, enabling quantitative detection even for weakly absorbing species [71]. Amplitude and linewidth-based schemes respond to changes in total damping and favor simplicity and speed, although they often require careful baseline stabilization [72]. In material-mediated approaches, chemical selectivity is provided by a responsive layer whose gas-induced optical changes are transduced by the MS into measurable signals such as intensity, phase, or polarization modulation [73].

MSs further enable non-spectral readout strategies by encoding gas-induced perturbations into polarization conversion or spatial wavefront modification [74]. These approaches allow direct visual or imaging-based detection and can operate without wavelength-resolved instrumentation. While such responses are often discrete or threshold-based rather than continuous, they demonstrate the flexibility of MSs as optical transducers that map chemical interaction onto diverse observables beyond conventional spectroscopy. Together, these considerations define a unified physical framework for flatland MS gas sensing based on resonance perturbation, energy redistribution, and field overlap. Table 1 organizes the principal transduction mechanisms according to their dominant perturbations, supported resonance types, and intrinsic trade-offs. This framework enables systematic comparison across material platforms and spectral regimes and provides a foundation for rational MS design tailored to specific sensing requirements.

## 3. Absorption and Spectroscopy-Based MS Gas Sensing

Absorption-based MS gas sensing has progressed beyond simple field-enhancement concepts toward system-level implementations in which MSs simultaneously perform multiple optical functions [85]. In particular, the ability of resonant MSs to act as spectrally selective emitters, absorbers, and detectors enables absorption spectroscopy to be realized within fully planar and highly integrated architectures [86]. By engineering both radiative and absorptive properties at the MS level, optical functionality can be confined to ultrathin surfaces while maintaining sufficient spectral selectivity, angular tolerance, and thermal efficiency for practical gas sensing [87]. This approach addresses key limitations of conventional nondispersive infrared systems, which rely on bulky emitters, external optical filters, and macroscopic optical paths, and establishes a route toward miniaturized, low-power, and filter-free spectroscopic sensors.

In this context, Lochbaum et al. reported a compact mid-infrared gas sensing platform in which all essential optical functionalities are realized using planar metamaterial structures, thereby eliminating conventional bulk optical components [86]. In this architecture, wavelength-selective emission and detection required for molecular absorption-based gas sensing are implemented using MS-engineered absorbers integrated on microelectromechanical membranes. Operation in the mid-infrared spectral region corresponding to the fundamental vibrational absorption of carbon dioxide enables true absorption spectroscopy while significantly reducing device footprint, optical path length, and power consumption compared with conventional nondispersive infrared gas sensors. The use of cascaded MS thermal emitters and MS detectors provides sufficient spectral selectivity and angular robustness without external optical filters, demonstrating that MSs can replace free-space optical components in practical gas sensing systems.

**Figure 2:**
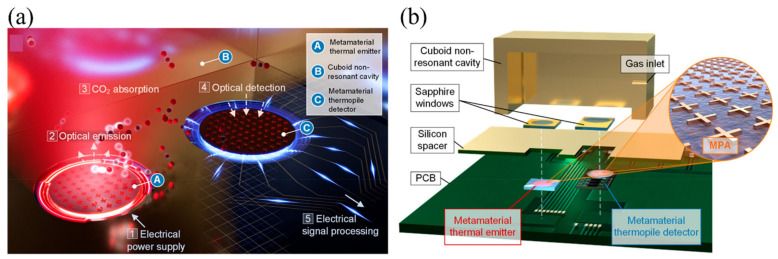
All-metamaterial mid-infrared gas sensor: (**a**) Operating principle of the MS-enabled gas sensor. An electrically driven metamaterial thermal emitter generates spectrally selective mid-infrared radiation that propagates through a compact non-resonant free-space cavity containing the target gas. Multiple reflections within the cavity increase the effective interaction length, and absorption by CO_2_ reduces the radiant power reaching the metamaterial thermopile detector, which converts the incident radiation into a thermoelectric voltage proportional to gas concentration. Wavelength selectivity is provided by metamaterial perfect absorber layers integrated on both the emitter and detector, eliminating the need for external optical filters. (**b**) Exploded view of the fabricated sensor prototype, showing the MS emitter and detector chips mounted on a printed circuit board and hermetically sealed from the non-resonant cavity using a silicon spacer and sapphire windows. The combined footprint of the emitter and detector is 5 × 5 mm^2^ [86].

Figure 2a illustrates the overall sensing concept and system architecture. An MS-based thermal emitter generates spectrally selective mid-infrared radiation that propagates through a compact gas cell containing the target analyte, after which the transmitted radiation is detected by an MS-integrated thermopile detector with a spectral response matched to the molecular absorption band of interest. Both the emitter and detector employ metamaterial perfect absorber designs, enabling emission, filtering, and detection to be implemented entirely using planar MSs rather than bulk interference filters, lenses, or optical cavities. This configuration exemplifies a flatland sensing approach in which optical functionality is confined to ultrathin surfaces while retaining sensitivity to gas-phase molecular absorption [86].

Figure 2b shows the operating principle of the MS thermal emitter, which consists of a patterned metamaterial absorber fabricated on a suspended membrane and exhibits high emissivity within a narrow spectral band defined by the MS geometry. Upon electrical heating, the structure emits mid-infrared radiation with a tailored spectral profile that overlaps the carbon dioxide absorption line. The authors further demonstrated that the MS emitter maintains stable spectral characteristics over a broad angular range, a requirement for reliable operation in compact gas sensing geometries. Together, the results associated with Figure 2a,b establish MSs as multifunctional optical elements that can simultaneously serve as sources, filters, and detectors, enabling absorption-based gas sensing within a miniaturized and energy-efficient flatland architecture [86]. In this system, CO_2_ absorption within the compact cavity produces a concentration-dependent reduction in detected radiant power, confirming that metamaterial spectral matching enables practical absorption-based gas sensing.

## 4. Refractive Index and Resonance Shift Sensing Mechanisms

Refractive index–based MS gas sensing exploits perturbations of the local electromagnetic environment induced by gas exposure rather than direct molecular absorption [88,89,90]. Variations in gas density, surface adsorption, or infiltration into functional or porous layers modify the effective refractive index sampled by the resonant near field, resulting in measurable shifts in resonance frequency or wavelength. This mechanism is particularly effective for weakly absorbing gases and for operation in spectral regions where detector technologies are readily available. Sensor performance is governed by the balance between resonance linewidth, field confinement, and environmental overlap, motivating MS designs that enhance sensitivity while maintaining robustness against optical loss, thermal drift, and fabrication variability [91,92].

Hybrid metal–dielectric MSs constitute an important class of refractive index sensing platforms, as they combine strong electromagnetic field confinement with reduced radiative loss [91]. In a representative numerical study, a metal–dielectric tetramer MS was investigated for refractive index sensing, where hybridized resonant modes arise from coupling between dielectric resonators and metallic elements. This structure exhibited a refractive index sensitivity of 500.94 nm/RIU, together with a quality factor of 793.13 and a figure of merit of 491.12 RIU^−1^, demonstrating high detection resolution enabled by sharp resonances and strong near-field localization. The symmetric tetramer configuration further provided polarization-robust sensing behavior, highlighting the suitability of hybrid metal-dielectric MSs for refractive index–based gas sensing without reliance on intrinsic molecular absorption [91].

An alternative strategy employs all-dielectric MSs supporting bound states in the continuum (BICs) to suppress radiative losses and achieve ultra-narrow resonances [93]. By introducing controlled symmetry breaking, symmetry-protected BICs can be converted into accessible quasi-BIC modes with high quality factors. In a dual-resonance all-dielectric MS studied for environmental refractive index sensing, quasi-BIC modes exhibited refractive index sensitivities decreasing from approximately 230 nm/RIU to 122 nm/RIU over the investigated refractive index range, while maintaining quality factors exceeding 400 for both resonances. The strong dependence of resonance position on the surrounding dielectric environment enables precise detection of small refractive index variations with enhanced spectral resolution compared with conventional dielectric resonators [93]. Beyond single-mode sensing, the same all-dielectric quasi-BIC platform demonstrated a dual-resonance differential sensing scheme, in which refractive index changes are inferred from variations in the spectral separation between two closely spaced resonant modes rather than from absolute wavelength shifts. This approach was shown to suppress sensitivity to environmental fluctuations such as temperature drift and instrumental instability, while preserving refractive index sensitivities comparable to those of single-resonance operation. These results illustrate how resonance multiplicity can be exploited to balance sensitivity and measurement robustness in practical refractive index sensing applications [93].

MS absorbers, while not always functioning directly as refractive index sensors, provide important building blocks for resonance-based sensing architectures [21,94,95]. For example, a narrowband perfect MS absorber based on impedance matching was numerically demonstrated using silicon meta-atoms patterned on a gold back reflector. This structure achieved absorption exceeding 95% at a resonance wavelength near 1137 nm and maintained high absorption over a broad range of incident angles [79]. Although refractive index sensitivity was not explicitly reported, the strong field localization and narrow resonance linewidth make such impedance-matched absorbers well suited for integration with functional layers, where environmental refractive index changes can be converted into measurable resonance shifts [79].

A representative numerical study of a hybrid plasmonic MS absorber for refractive index sensing was reported, in which dielectric silicon nitride meta-atoms are integrated with a metallic gold back reflector to support narrowband resonances that are highly sensitive to the surrounding dielectric environment [15]. The results show that planar MS absorbers can function as efficient refractive index sensors by tracking resonance wavelength shifts induced by changes in the local refractive index, without relying on molecular absorption features. By engineering resonance linewidth and electromagnetic field confinement, large wavelength shifts per refractive index unit and high figures of merit are achieved, highlighting the suitability of resonance-shift-based sensing for weakly absorbing gases or analytes in detector-accessible spectral regions [20].

To clarify the role of the resonance quality factor in sensing performance, two representative MS configurations can be considered. Square silicon nitride meta-atoms support spectrally narrow resonances with moderate quality factors and exhibit polarization- and angle-insensitive responses, providing robustness against variations in illumination conditions. In contrast, cross-slot meta-atoms support quasi-bound states in the continuum, which suppress radiative losses and produce ultra-high-Q resonances. These quasi-BIC modes yield substantially enhanced refractive index sensitivity, as small perturbations of the surrounding dielectric environment result in clearly resolvable resonance shifts, albeit with increased sensitivity to fabrication tolerances.

**Figure 3:**
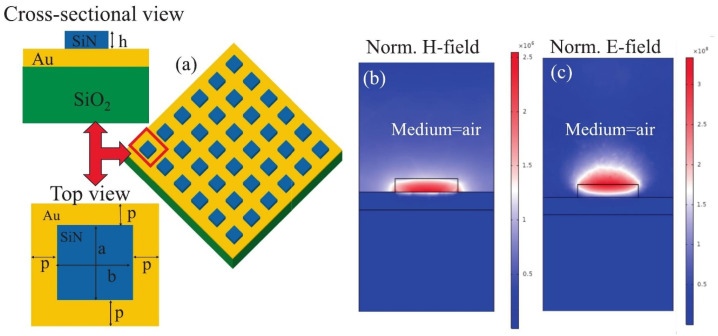
Hybrid plasmonic MS architecture: (**a**) Three-dimensional schematic of the hybrid plasmonic MS absorber. The inset shows the two-dimensional top-view geometry of the MS unit cell. Normalized electromagnetic field distributions at the resonance wavelength are shown for (**b**) the magnetic field and (**c**) the electric field [20].

The sensing mechanism is illustrated in Figure 3. The unit-cell schematic in Figure 3a shows a silicon nitride meta-atom patterned on a continuous gold film supported by a dielectric substrate. The gold layer acts as an optically opaque back reflector that enforces strong coupling between the incident field and the MS resonance, while the silicon nitride element functions as a low-loss dielectric resonator defining the resonance wavelength. The magnetic field distribution at resonance in Figure 3b exhibits strong localization at the silicon nitride–gold interface, indicating a hybrid magnetic resonance formed by coupling between a dielectric Mie-type magnetic dipole and image currents in the metal. The corresponding electric field distribution in Figure 3c is mainly confined within the silicon nitride meta-atom, with partial extension into the surrounding medium, providing the near-field overlap required for refractive index sensing. Together, these field profiles illustrate how hybrid dielectric–plasmonic resonances combine narrow linewidths with strong environmental sensitivity, enabling high refractive index sensing performance [20]. In gas sensing applications, such resonance perturbations enable quantitative detection by converting gas-induced refractive index variations or adsorption effects into measurable wavelength shifts with ppm-level detection capability depending on resonance linewidth and field overlap.

## 5. Loss Damping and Amplitude Modulation-Based Detection

In addition to resonance frequency shifts, gas exposure can modify the damping rate of MS resonances, leading to measurable changes in transmission amplitude, reflection contrast, or resonance linewidth [96]. These effects occur when gaseous analytes introduce additional loss channels through absorption or scattering, altering the balance between radiative and dissipative decay processes. From a resonance physics perspective, gas-induced damping reduces photon lifetime and effective quality factor, translating chemical interaction into amplitude or contrast modulation rather than spectral displacement [97]. MSs that rely on plasmonic resonances are particularly sensitive to these loss effects due to the intrinsic absorption of metallic elements, which can amplify damping perturbations but also increase baseline loss [85].

Amplitude-based detection schemes offer practical advantages over wavelength tracking because they can be implemented at a fixed interrogation wavelength using simple photodetectors rather than spectrally resolving instruments [98]. This enables compact system architectures and supports high-speed readout, which is attractive for real-time monitoring and threshold-based sensing scenarios where rapid signal changes are sufficient for detection. The ability of MSs to tailor amplitude response through resonant absorption and interference effects has been highlighted in recent surveys of MS sensor designs spanning gigahertz to optical frequencies [99].

However, distinguishing gas-induced loss from intrinsic material absorption, surface roughness, and fabrication variability remains a central challenge. Because amplitude modulation depends on the absolute balance of loss channels, baseline drift and environmental fluctuations can significantly affect signal fidelity. Accurate interpretation, therefore, requires careful calibration and, in some implementations, differential referencing or complementary sensing modalities to suppress spurious contributions. Reviews of MS design principles note that amplitude modulation is intertwined with absorption and interference mechanisms, and that control of these effects is key to reliable sensing performance across diverse spectral regimes [39].

Despite these limitations, damping and amplitude modulation-based approaches provide a practical sensing pathway in applications where simplicity, speed, and system integration are prioritized over absolute chemical specificity or high spectral resolution [100]. Their use complements other transduction strategies such as refractive index shifts or molecular absorption enhancement, expanding the toolkit available for MS-based gas sensing platforms.

**Figure 4:**
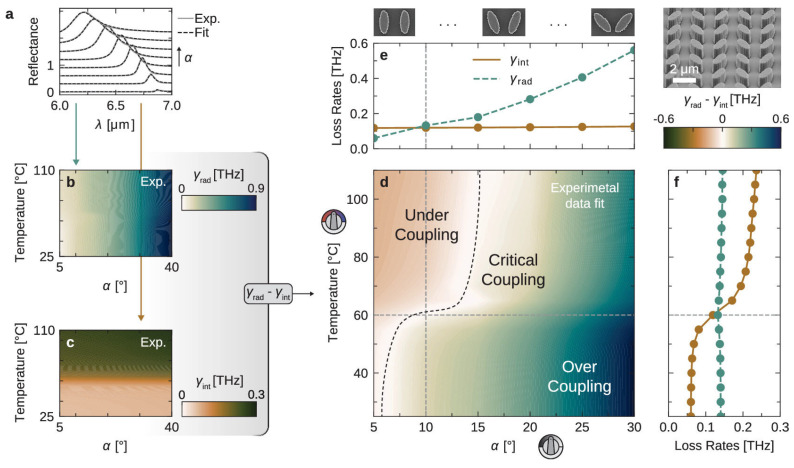
Temporal coupled mode theory (TCMT) analysis of loss-controlled resonance behavior: (**a**) Measured reflectance spectra (gray) and TCMT fits (dashed) at 25 °C for asymmetry angles α = 5°–40°. (**b**,**c**) Extracted radiative (γ_rad_) and intrinsic (γ_int_) loss rates, showing that γ_rad_ increases with α and is temperature-independent, whereas γ_int_ increases with temperature and is insensitive to α. (**d**) Map of γ_rad_ − γ_int_ identifying undercoupled, critically coupled, and overcoupled regimes. (**e**,**f**) Cross-sections of γ_rad_ and γ_int_ versus asymmetry (60 °C) and temperature (α = 10°), demonstrating independent control of radiative and intrinsic losses [100].

Within this framework, the loss-damping concepts outlined above are directly supported by recent experimental work on actively loss-engineered MSs, as summarized in Figure 4a–f [100]. Rather than relying on resonance frequency shifts, this study demonstrates that deliberate modification of loss channels can serve as the primary transduction mechanism. By integrating a thin vanadium dioxide layer into a high-quality-factor bound-state-in-the-continuum MS, the authors show that increasing intrinsic absorption predominantly shortens photon lifetime and reduces the quality factor, resulting in pronounced changes in resonance amplitude and contrast while leaving the resonance frequency largely unchanged. This response closely parallels the sensing scenario discussed in this section, where gas adsorption or molecular interaction introduces additional dissipative channels that manifest as amplitude modulation rather than spectral displacement [100].

Figure 4a illustrates this behavior by comparing measured reflectance spectra with temporal coupled-mode theory fits, confirming that the observed amplitude suppression and linewidth broadening arise from controlled changes in resonance damping rather than uncontrolled spectral distortions. Figure 4b,c explicitly separate the two decay pathways governing the response: the radiative loss rate is determined solely by structural asymmetry and remains independent of temperature, whereas the intrinsic loss rate increases strongly with temperature due to the VO_2_ phase transition. This clear decoupling provides direct experimental validation of the balance between radiative and dissipative decay processes discussed earlier and demonstrates how amplitude-based signals originate from changes in intrinsic loss at a fixed interrogation wavelength [100].

The combined impact of these loss channels is visualized in Figure 4d, which maps the transition between undercoupled, critically coupled, and overcoupled regimes as the relative magnitudes of radiative and intrinsic losses evolve. This representation highlights why amplitude modulation is maximized near critical coupling and clarifies why damping-based detection schemes are inherently sensitive to absolute loss levels, consistent with the calibration and stability challenges noted above. Finally, Figure 4e,f show that a single MS can be continuously driven across these coupling regimes by tuning either geometry or intrinsic absorption, resulting in large and reversible amplitude modulation. Together, these results form a coherent experimental bridge between the resonance-physics arguments presented in this section and practical implementations of loss- and amplitude-modulation-based MS sensing [100].

## 6. Material-Mediated and Chemo-Optical Flatland Sensors

Material-mediated MS sensors incorporate functional layers whose optical properties change in response to gas exposure. Liquid crystals, metal oxides, graphene, and other responsive materials can be integrated with MSs to transduce chemical interactions into refractive index modulation, polarization rotation, or amplitude variation [101,102,103,104]. In such systems, the MS serves primarily as an optical amplifier and readout interface rather than as the dominant interaction medium [105]. Chemo-optical approaches offer enhanced sensitivity and tunability through material engineering, but often sacrifice universality and long-term stability [106,107,108]. Response time, reversibility, and selectivity are governed by diffusion kinetics and chemical binding processes rather than purely electromagnetic effects. Consequently, these platforms are best suited for application-specific sensing scenarios in which target gases and operating conditions are well defined.

**Figure 5:**
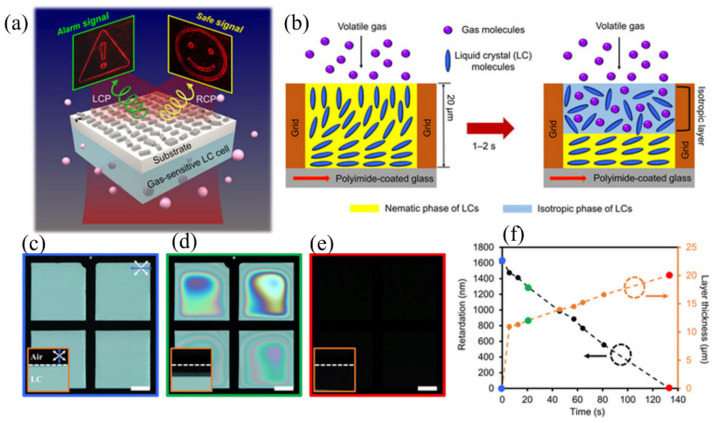
Gas-responsive liquid crystal MS sensor: (**a**) Holographic MS integrated with a gas-responsive liquid crystal layer that displays polarization-dependent safety and alarm signals. (**b**) Liquid crystal cell geometry and gas sensing mechanism, where gas diffusion reduces orientational order and induces a nematic-to-isotropic transition. (**c**–**e**) Optical micrographs showing time-dependent liquid crystal disordering during isopropyl alcohol exposure. (**f**) Corresponding evolution of optical retardation and isotropic layer thickness [73].

A representative example of this material-mediated flatland sensing paradigm was demonstrated by Zhang et al. [73], who explicitly decoupled the chemical interaction mechanism from the electromagnetic response of the MS. In this work, the authors deliberately decoupled the chemical interaction mechanism from the electromagnetic response of the MS. Gas molecules did not directly perturb MS resonances through absorption or refractive index modulation. Instead, chemical sensitivity originated in the liquid crystal layer, while the MS functioned as a planar optical element that converted material-induced polarization changes into a visually discernible holographic response. The overall device architecture and sensing concept are illustrated in Figure 5a, where the MS is positioned beneath a thin liquid crystal layer to form an ultrathin planar sensing stack. Incident light first propagates through the liquid crystal before interacting with the MS, ensuring that gas-induced modifications of the liquid crystal directly influence the optical state of the incident field. The authors emphasized that the sensing and readout processes occur entirely within a subwavelength thickness, consistent with a flatland optical interaction regime rather than a volumetric propagation-based approach [73].

The gas sensing mechanism is schematically described in Figure 5b. Upon exposure to volatile gas molecules, diffusion into the liquid crystal layer reduces the orientational order of the nematic phase, leading to a partial or complete transition toward an isotropic state near the interface. This molecular reordering decreases birefringence and modifies the optical retardation of the liquid crystal layer. As a result, the polarization state of transmitted light is altered. The authors demonstrated that this response is governed by chemo-optical interactions and diffusion kinetics rather than by electromagnetic coupling between gas molecules and the MS itself. The MS design and optical transduction principle are shown in Figure 5c. The dielectric MS was engineered to exhibit polarization-dependent holographic wavefront reconstruction, such that different polarization states of incident light produce distinct holographic images. In the absence of gas exposure, the ordered liquid crystal preserves the initial polarization state, and the MS reconstructs a predefined hologram. Gas-induced polarization conversion activates an alternative holographic response, enabling the MS to encode chemical information into spatial wavefront modulation [73].

Experimental holographic switching is presented in Figure 5d, where distinct holographic images are observed before and after gas exposure. The authors reported that the transition between holographic states occurs abruptly once a threshold level of liquid crystal disorder is reached. This behavior results in a binary or threshold-based sensing response, which differs fundamentally from continuous spectral or resonance shift-based detection schemes. The spatial nature of the readout eliminates the need for wavelength-resolved measurements or complex signal processing. The temporal characteristics of the sensing response are summarized in Figure 5e. The response time was found to be governed primarily by gas diffusion into the liquid crystal layer and subsequent relaxation of molecular ordering. The authors showed that the process is reversible under controlled conditions, indicating that the sensing mechanism relies on a reversible physical transition rather than an irreversible chemical reaction. However, they also noted that response speed and selectivity are intrinsically linked to the properties of the liquid crystal and the chemical nature of the gas species. System-level integration and application potential are demonstrated in Figure 5f, where the MS sensor was fabricated on a flexible substrate and incorporated into a wearable configuration. This demonstration highlights the compatibility of flatland MS sensors with conformal and lightweight platforms and underscores their suitability for user-facing and safety-related applications. The authors emphasized that the instrument-free visual readout enabled by holographic wavefront encoding represents a practical advantage for scenarios where rapid and intuitive gas detection is required [73].

Overall, this work establishes a distinct class of flatland MS gas sensors in which chemical interaction, optical transduction, and readout functionality are spatially and functionally separated. By demonstrating that MSs can operate as programmable optical interfaces rather than direct sensing elements, the study broadens the scope of MS-enabled gas sensing beyond spectroscopic and resonance-based paradigms. The approach complements absorption and refractive index-based MS sensors and illustrates the versatility of flatland MSs as optical transducers for material-mediated chemical sensing [73].

## 7. Polarization Wavefront and Visual Readout Sensors

MSs enable gas sensing concepts based on changes in polarization state, phase profile, or reconstructed wavefront rather than spectral modulation. Gas-induced perturbations can trigger polarization conversion, holographic image switching, or intensity redistribution, providing intuitive visual or imaging-based readout without complex spectral analysis. These approaches are particularly attractive for wearable, safety, and user-facing applications. While wavefront-based sensing offers simplicity and immediacy, it typically provides limited quantitative information and reduced chemical specificity. Detection thresholds are often binary or qualitative, and selectivity depends strongly on auxiliary materials or functional layers. Nevertheless, such platforms highlight the versatility of MSs as transducers that map chemical interaction into diverse optical observables.

The work by Duan et al. demonstrates a gas-responsive plasmonic MS in which chemical interaction is transduced directly into a macroscopic, visually observable optical response, rather than a subtle spectral shift [109]. The MS consists of periodic arrays of magnesium (Mg) nanoparticles capped with Ti/Pd layers, where Mg acts as the active plasmonic material, and Pd catalyzes hydrogen dissociation. Exposure to hydrogen induces a reversible metal–dielectric phase transition (Mg → MgH_2_), fundamentally altering the plasmonic resonances of the MS and enabling dynamic modulation of colour, intensity, and image content in real time.

**Figure 6:**
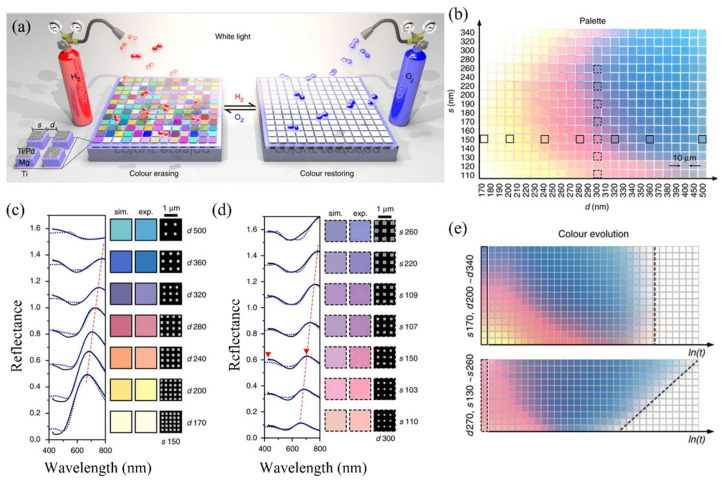
(**a**) Schematic of a hydrogen-responsive plasmonic MS composed of Mg nanoparticles capped with Ti/Pd, where exposure to hydrogen induces a reversible Mg–MgH_2_ phase transition that erases structural colors, while oxygen restores them. The nanoparticles are arranged in periodic color pixels defined by particle size *s* and spacing *d*. (**b**) Experimental color palette obtained by systematically varying *s* and *d*. (**c**,**d**) Corresponding experimental and simulated reflectance spectra and colors for selected pixels, showing geometry-dependent plasmonic resonances and reflectance peak shifts. (**e**) Time-resolved color evolution of representative pixels during hydrogen exposure, illustrating dynamic color fading and disappearance governed by hydrogenation kinetics [109].

Figure 6 summarizes the operating principle and the optical response of the dynamic plasmonic color display. Figure 5a schematically illustrates the sensing mechanism: incident white light is reflected by an MS composed of Mg nanoparticles arranged in a periodic lattice, producing vivid structural colors. Upon exposure to hydrogen, Mg absorbs hydrogen atoms (catalytically activated by the Pd layer) and transforms into MgH_2_, which is dielectric. This transition suppresses the plasmonic resonances responsible for color generation, leading to color erasing. Subsequent exposure to oxygen reverses the process, restoring metallic Mg and the original optical response. Figure 6b presents an experimental color palette obtained by systematically varying the nanoparticle size and interparticle spacing, demonstrating that a broad range of colors can be encoded purely through MS geometry.

Figure 6c,d correlate these colors with measured and simulated reflectance spectra, showing that each color arises from well-defined plasmonic resonances whose spectral positions depend on the lattice parameters. These panels establish the link between nanoscale design, resonant optical response, and perceived color. Figure 6e captures the temporal evolution of selected color pixels during hydrogen exposure, revealing the dynamic nature of the gas–MS interaction. As hydrogenation progresses, the reflectance spectra flatten, and the colors fade until they disappear entirely, with characteristic erasing times that depend on nanoparticle size and spacing. This time-resolved behavior highlights that the gas-induced optical response is governed not only by equilibrium material properties but also by hydrogen diffusion and phase-transition kinetics within the MS [109]. Overall, the findings demonstrate that gas exposure can be mapped onto large, intuitive visual changes through MS engineering, enabling a form of chemical sensing based on wavefront and intensity modulation rather than conventional spectroscopic analysis. The paper establishes magnesium-based plasmonic MSs as a versatile platform for gas-triggered visual readout, with implications for user-facing sensing, safety indicators, and adaptive optical devices.

## 8. Spectral Regimes and Operational Wavelengths

The operating wavelength fundamentally governs MS gas sensing performance by determining the dominant light–matter interaction mechanisms, intrinsic material losses, and compatibility with sources and detectors. Visible and near-infrared MS platforms benefit from mature nanofabrication and optoelectronic technologies [110]; however, because most gas molecules exhibit weak absorption in these spectral regions, sensing typically relies on indirect transduction mechanisms mediated by functional materials or refractive index perturbations [111]. Mid-infrared MSs provide direct access to molecular vibrational fingerprints, enabling chemically selective detection, but often face limitations associated with material absorption, thermal background radiation, and increased system complexity [112]. Terahertz MSs probe molecular rotational responses and dielectric perturbations with high chemical specificity and reduced interference from condensed-phase materials, albeit at the cost of larger device dimensions and specialized instrumentation [113]. Each spectral regime, therefore, presents a distinct balance between sensitivity, selectivity, scalability, and system complexity, which MS architectures can partially mitigate through engineered resonances and material selection.

In the terahertz regime, He et al. proposed a polarization-insensitive metallic MS based on asymmetric cross-shaped apertures that supports dual quasi-bound states in the continuum [57]. By deliberately breaking in-plane symmetry, their design enables excitation of high-Q quasi-BIC resonances originating from electric dipole and electric quadrupole modes. These resonances exhibit exceptionally high refractive index sensitivities, exceeding several hundred gigahertz per refractive index unit, and enable low-concentration carbon dioxide detection when combined with a polyhexamethylene biguanide (PHMB) functional layer. This work demonstrates how terahertz MSs can achieve high sensitivity through dielectric perturbation sensing, even in regimes where direct molecular absorption is weak [57].

In the infrared domain, MS gas sensors encompass both passive and actively tunable implementations. Kazanskiy et al. numerically demonstrated an MS perfect absorber composed of silicon nano-cylinders on a metallic back reflector, achieving polarization- and angle-insensitive narrowband absorption [45]. When coated with a PHMB layer, their MS translated carbon dioxide-induced refractive index changes into pronounced resonance wavelength shifts, enabling quantitative detection over several hundred parts per million. This work illustrates how impedance-matched MS absorbers can serve as compact alternatives to conventional optical filters in nondispersive infrared gas sensing systems [45].

Beyond passive operation, active tunability has been introduced to infrared MS gas sensors through material phase transitions. In a tunable dual-gas sensor proposed by Chen et al., vanadium dioxide was incorporated into a multilayer metal–dielectric–metal MS to exploit its insulator-to-metal phase transition [58]. By thermally switching the conductivity state of vanadium dioxide, distinct plasmonic resonance modes were selectively excited, enabling wavelength-switchable detection of methane and hydrogen within a single device. This approach highlights how phase-change materials can be leveraged to achieve multi-gas functionality without increasing structural complexity [58].

Mechanical reconfiguration provides an alternative route to tunability in the infrared regime. Li et al. demonstrated a tunable meta-absorber integrated with a microelectromechanical electrothermal actuator, in which a planar metal–insulator–metal cavity exhibits angle-dependent absorption [114]. By electrically controlling the inclination angle of the MS, the absorption resonance can be dynamically aligned with the characteristic absorption bands of different gases, enabling multi-gas nondispersive infrared sensing using a single absorber structure. This micro-electromechanical system (MEMS)-based strategy addresses miniaturization and integration challenges associated with conventional multi-filter non-dispersive infrared (NDIR) systems [114].

At shorter wavelengths, alternative transduction mechanisms dominate due to limited intrinsic molecular absorption [115]. In the visible regime, MSs are frequently employed as optical transducers that amplify gas-induced changes in functional materials, converting chemical interactions into measurable resonance shifts or polarization variations. In the deep-ultraviolet regime, plasmonic sensing approaches exploit the strong electronic absorption cross-sections of gases such as ozone and ammonia, enabling low-concentration detection relevant to environmental monitoring and high-voltage equipment protection. Although fabrication tolerances and material stability impose additional challenges at these wavelengths, access to intrinsic electronic transitions provides enhanced sensitivity for specialized applications [115].

To contextualize the spectral strategies discussed above, material selection must be considered in relation to the target operating wavelength. In flatland MS gas sensing, the choice of constituent materials governs the attainable field confinement, resonance quality factor, and operational bandwidth, thereby setting wavelength-dependent performance limits [78,116]. Plasmonic metals provide strong near-field enhancement in the visible and infrared regimes but incur significant ohmic losses, whereas high-index dielectric MSs enable low-loss resonances that are particularly advantageous at longer wavelengths [20]. Hybrid metal–dielectric and polaritonic platforms offer intermediate solutions by redistributing electromagnetic energy to balance sensitivity and quality factor across different spectral bands [116]. Functional material overlays further introduce chemical selectivity, with trade-offs in response speed and long-term stability that become increasingly important in broadband and multispectral sensing architectures [45].

In practice, integrating 2D materials and porous frameworks into MS platforms introduces materials-level constraints that often dominate sensor dynamics [117]. For graphene and related 2D layers, adsorption/desorption kinetics and charge-transfer equilibration processes set the response and recovery times, while humidity, contamination, and defect evolution can degrade stability over repeated cycles [118]. For MOFs, strong adsorption can improve sensitivity and selectivity but may slow desorption and complicate regeneration, particularly for strongly binding species or in humid environments where competitive adsorption occurs [119]. A further bottleneck is achieving uniform, conformal coatings on nanostructured MS topographies; non-uniform thickness and incomplete coverage introduce spatially varying analyte overlap and additional optical loss [120]. Scalable integration therefore requires controlled deposition and growth methods that ensure conformality and robustness, coupled with packaging strategies that stabilize the functional layer under realistic operating conditions.

Table 2 summarizes the principal material classes employed in flatland MS gas sensing, detailing their electromagnetic response, supported resonant modes, dominant spectral regimes, and functional roles within sensing architectures. By explicitly identifying both advantages and inherent limitations, this comparison clarifies how spectral regime and material choice jointly mediate the trade-offs between sensitivity, selectivity, loss, and system complexity.

## 9. MS Architectures and Design Strategies

**Figure 7:**
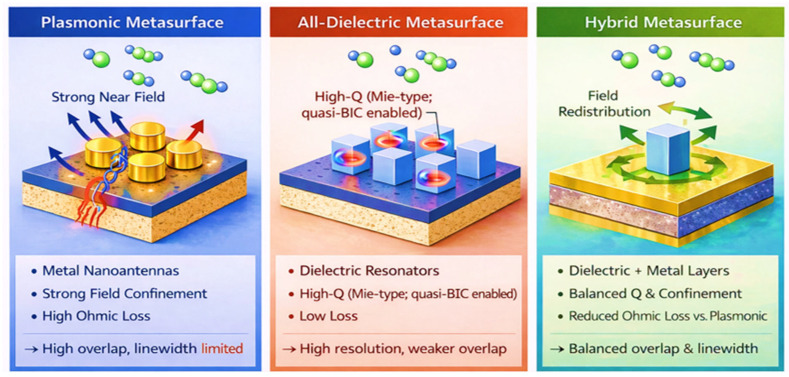
Schematic comparison of plasmonic (**left**), all dielectric (**middle**), and hybrid MS architectures (**right**) for gas sensing. The figure highlights representative structural concepts, qualitative electromagnetic field localization, and dominant loss mechanisms that govern sensing performance across different MS platforms.

Figure 7 presents a schematic comparison of the three principal MS architectures employed in gas sensing, namely plasmonic, all dielectric, and hybrid platforms. The figure highlights their characteristic field distributions, dominant loss mechanisms, and sensing relevant trade-offs, providing a unified architectural perspective for interpreting the diverse design strategies discussed in this section. Rather than emphasizing a single performance metric, this comparison illustrates how architectural choice governs the balance between near-field enhancement, resonance linewidth, and analyte field overlap, which ultimately determines sensitivity, resolution, and robustness in practical sensing systems. These structural differences directly translate into gas sensing performance trade-offs, where plasmonic architectures provide strong sensitivity via field confinement, dielectric architectures enable higher resolution through narrow resonances, and hybrid platforms balance sensitivity and loss for practical gas detection.

Plasmonic MSs achieve extreme field confinement and strong interaction with gaseous analytes, but are fundamentally limited by ohmic losses that broaden resonances and reduce spectral efficiency [67,136]. All-dielectric MSs mitigate these losses by supporting high-quality-factor resonances based on Mie-type modes and interference effects, enabling sharper spectral features and improved sensing resolution [71,137]. Hybrid architectures combine plasmonic and dielectric elements or integrate functional materials to leverage complementary advantages, enabling multimodal sensing, tunability, and enhanced selectivity at the cost of increased fabrication complexity [129,135,138,139]. The choice of MS architecture must therefore be guided by application-specific requirements rather than a single performance metric.

**Figure 8:**
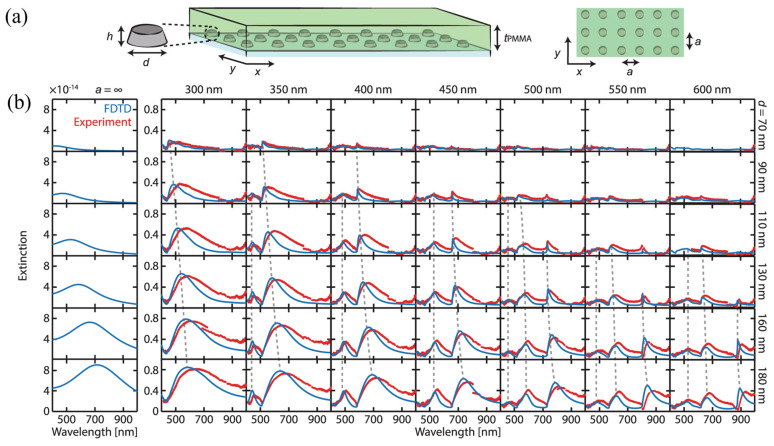
(**a**) Schematic illustration of the plasmonic MS sample. (**b**) Experimental (red) and simulated (blue) extinction spectra measured at normal incidence for square arrays of palladium nanodisks with a fixed height of 45 nm and a polymer overlayer thickness of 200 nm. The lattice period increases from 300 to 600 nm along the horizontal axis, while the nanodisk diameter varies from 70 to 180 nm along the vertical axis. The leftmost panels show the extinction cross sections of the corresponding isolated nanodisks, representing the limit of negligible interparticle coupling. Compared to isolated particles, periodic arrays exhibit distinctly modified optical responses, with narrow resonances originating from the hybridization of localized surface plasmon modes with Rayleigh anomaly modes, forming surface lattice resonances. The dashed gray lines indicate the systematic evolution of these resonances with increasing nanodisk diameter [140].

The inverse-designed plasmonic MS based on periodic palladium nanoparticle arrays demonstrates both the strengths and intrinsic limitations of purely plasmonic architectures for gas sensing [140]. By arranging lossy Pd nanodisks into a two-dimensional periodic lattice, the study shows that collective surface lattice resonances can substantially narrow spectral linewidths compared to isolated nanoparticles, thereby improving sensing resolution. This architectural principle is illustrated in Figure 8a, where the schematic highlights the periodic array geometry embedded in a dielectric environment, emphasizing that the sensing platform relies entirely on metallic nanostructures. The corresponding extinction spectrum in Figure 8b reveals the emergence of narrow collective resonances that differ qualitatively from the broad localized surface plasmon resonances of single particles. These results establish that architectural control through lattice effects can partially compensate for radiative losses in plasmonic systems. However, the field distributions and linewidths also make clear that a significant fraction of the electromagnetic energy remains confined within the metal, where ohmic losses fundamentally limit the achievable quality factor. As a result, optimal performance arises from a trade-off between resonance narrowing and sufficient field enhancement inside the metallic nanostructures, rather than from maximizing either quantity independently [140]. Such lattice-engineered plasmonic structures have been experimentally applied to gas sensing, where gas adsorption modifies the dielectric environment and produces measurable extinction spectral shifts that scale with gas concentration.

**Figure 9:**
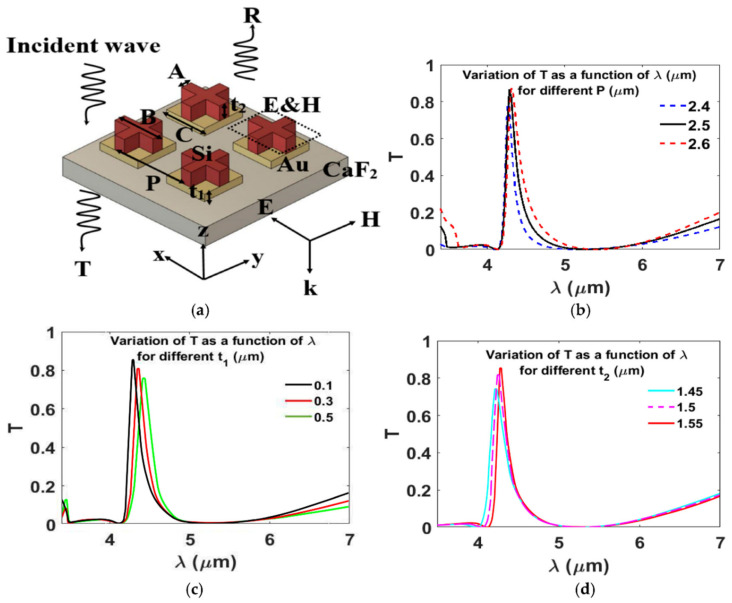
(**a**) Schematic of the MS filter with key geometrical parameters, including the period P, gold thickness t_1_, silicon thickness t_2_, cross dimensions A and B, and gold patch length C. (**b**–**d**) Simulated transmission spectra showing the effect of varying (**b**) the period P, (**c**) the gold thickness t_1_, and (**d**) the silicon thickness t_2_ on the transmission efficiency T, with all other parameters fixed. The optimized design uses P = 2.5 μm, A = 0.3 μm, B = 2.2 μm, C = 2.2 μm, t_1_ = 0.1 μm, and t_2_ = 1.55 μm [84].

Hybrid metal-dielectric MSs address these limitations by redistributing electromagnetic energy away from lossy metals while preserving strong light–matter interaction. In the hybrid architecture reported by Soliman et al., dielectric resonators are combined with metallic layers to form a composite MS that separates resonance formation from loss mechanisms [84]. This design strategy is summarized in Figure 9a, which shows the hybrid unit cell composed of dielectric elements integrated with a metallic layer, clearly contrasting with the fully metallic architecture of the plasmonic array. Figure 9b–d further illustrate how this hybridization modifies the optical response: the spectra demonstrate sharper and more controllable resonances, while the corresponding field distributions reveal that a substantial portion of the electromagnetic energy is stored within the dielectric components rather than dissipated in the metal. This redistribution reduces ohmic damping and enables higher spectral efficiency, while the metallic layer continues to provide impedance matching, absorption control, or spectral selectivity [84].

Together, these two studies highlight architectural progression in MS design. Purely plasmonic MSs rely on collective lattice effects, as evidenced in Figure 9a,b of the plasmonic array, to mitigate radiative losses but remain constrained by intrinsic material absorption [140]. Hybrid MSs, as shown in Figure 9a–d of the metal–dielectric platform, introduce additional design degrees of freedom that decouple field confinement from loss, enabling sharper resonances and improved robustness at the expense of increased structural complexity [84]. This comparison underscores that MS architecture plays a decisive role in defining performance limits and practical trade-offs, and that the choice between plasmonic and hybrid platforms should be guided by application-specific requirements rather than a single sensing metric.

## 10. System-Level Integration and Applications

For MS gas sensors to transition from laboratory demonstrations to practical deployment, system-level integration is a decisive requirement. The planar nature of MSs enables direct compatibility with complementary metal-oxide-semiconductor (CMOS) processes, on-chip photodetectors, microbolometers, and readout electronics, allowing sensing, filtering, and spectral selectivity to be co-integrated within compact modules. Recent CMOS–SOI–MEMS implementations demonstrate that MS absorbers and filters can be monolithically integrated with thermal detector pixels to achieve spectrally selective, near-unity absorption at gas-relevant wavelengths, while preserving fabrication compatibility and device scalability [95].

**Figure 10:**
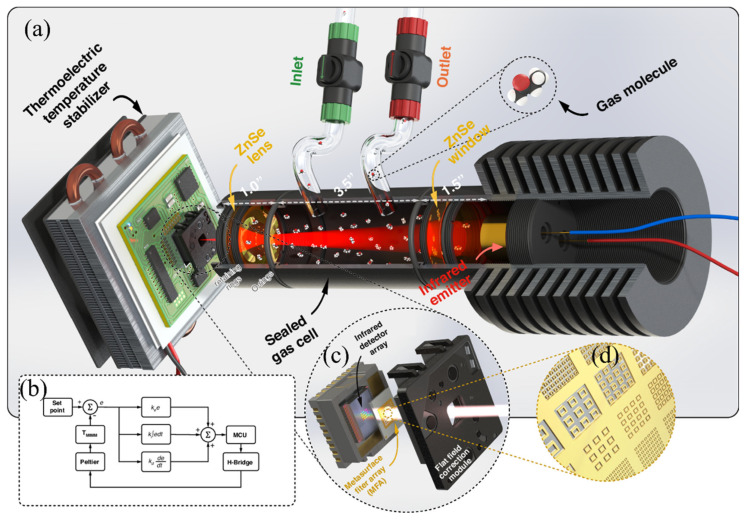
Integrated MS gas sensing system: (**a**) Schematic of the system, with a MS infrared microspectrometer (MIMM) and infrared emitter placed on opposite sides of a sealed gas cell; the MIMM is mounted on a thermoelectric temperature stabilizer. (**b**) Closed-loop thermoelectric temperature stabilization (TTS) control scheme. (**c**) MIMM architecture showing an MS spectral filter array bonded to a microbolometer camera for direct spectral encoding and detection. (**d**) Schematic of the metallic MS filter array defining the spectral response through subwavelength patterning [42].

A representative system-level realization is the smart mid-infrared MS microspectrometer (MIMM) reported by Meng et al., which illustrates how MSs can function as enabling optical interfaces within a complete gas sensing system rather than as isolated components [42]. As shown in Figure 10a, the system integrates an MS-based microspectrometer, a broadband infrared emitter, a sealed gas cell, and electronic temperature control into a compact, portable architecture. Infrared radiation transmitted through the gas cell is spectrally encoded by the MS filter array and directly detected by an underlying microbolometer array, eliminating the need for bulky dispersive optics or moving parts. Figure 10b details the closed-loop thermoelectric temperature stabilization scheme, highlighting the importance of thermal control at the system level to suppress drift in thermal detector readout. Figure 10c illustrates the filter-array–detector-array (FADA) architecture, where a planar MS spectral filter chip is bonded directly onto a commercial microbolometer camera, demonstrating seamless optical and electrical integration.

Finally, Figure 10d shows the MS unit-cell geometry used to realize bandpass and bandstop responses, emphasizing that spectral selectivity is encoded purely through planar nanostructure design. Beyond architectural integration, this work establishes several key system-level findings: (i) MS spectral filtering enables compact mid-IR spectroscopy within volumes on the order of cubic centimeters; (ii) thermal stabilization is essential to maintain signal fidelity in MS–microbolometer systems; and (iii) MS-enabled spectral encoding can be combined with data-driven readout strategies, such as machine learning, to enable multigas identification without wavelength scanning or full spectral reconstruction. Together, these results demonstrate that MSs can serve as scalable, manufacturable interfaces between optical transduction and electronic readout, bridging the gap between nanophotonic design and deployable gas sensing systems.

Effective integration more generally requires coordinated optimization of optical coupling, thermal behavior, mechanical stability, and packaging. Optical–thermal co-design frameworks link MS spectral efficiency to absorbed power and detector temperature rise, enabling predictive system-level optimization. At the same time, MS integration introduces trade-offs, such as increased thermal capacitance and modified time constants, which must be balanced against responsivity and bandwidth requirements. Environmental isolation through wafer-level vacuum packaging is critical to suppress parasitic heat transfer and ensure stable operation, while mechanical considerations such as mass loading and residual stress must be managed to preserve robustness and alignment in integrated platforms [42].

In practical manufacturing, these system-level constraints translate directly into fabrication yield and thermal management requirements. For high-quality-factor dielectric and quasi-BIC MSs, resonance linewidths can fall below approximately 1 nm in the optical domain, meaning that dimensional variations on the order of 5–20 nm can introduce measurable resonance shifts and device-to-device spectral variability [141]. Maintaining such tolerances over wafer-scale fabrication typically requires advanced lithographic control or post-fabrication calibration, which can directly influence manufacturing yield and per-device cost [142]. In contrast, plasmonic MSs generally exhibit broader resonances (typically tens of nanometers), making them more tolerant to geometric variation but with reduced spectral resolution [143].

Thermal stabilization requirements also differ across platforms. Plasmonic MSs can often tolerate temperature fluctuations of several Kelvin without significant signal degradation, whereas high-Q dielectric and BIC-based MSs used for quantitative sensing may require temperature stability within approximately 0.1–1 K [144]. These differences directly impact packaging complexity, power consumption, and overall system cost, particularly in portable or wearable sensing implementations.

Applications of MS gas sensors include environmental monitoring, industrial safety, medical diagnostics, and wearable platforms. The selection of flatland MS sensing mechanisms for specific applications is primarily governed by system-level performance priorities rather than intrinsic sensitivity alone. Quantitative monitoring tasks, such as environmental sensing and industrial process control, typically favor resonance-shift or spectrally resolved detection due to their stability and calibration compatibility [145]. In contrast, safety-critical and wearable sensing scenarios prioritize rapid response, low power consumption, and minimal instrumentation, favoring amplitude-based or visually encoded readout strategies [146]. Applications requiring molecular specificity, including medical diagnostics and trace-gas analysis, benefit from spectrally selective absorption-based detection, despite increased system complexity. In practice, platform selection reflects trade-offs among selectivity, response speed, system complexity, and deployment cost, rather than optimization of a single sensing metric [147].

Compact NDIR-style gas modules benefit from MS-enabled spectral selectivity and on-chip detection, enabling reduced footprint and power consumption [148]. Flexible and conformal MS implementations further extend sensing capabilities to curved, portable, and user-facing systems [73]. More broadly, the ability to engineer both the sensing mechanism (e.g., absorption, resonance shift, or chemo-optical modulation) and the readout modality (spectral, thermal, or visual) positions MS gas sensors as adaptable platforms for diverse operational contexts [86].

## 11. Challenges Outlook and Conclusions

Flatland MSs define a forward path for optical gas sensing by shifting the governing interaction from volumetric light propagation to surface-confined resonant physics. The key scientific implication is that gas detection in such systems is determined by how perturbations modify the complex eigenstates of planar optical modes, rather than by interaction alone. This insight enables a unified understanding of diverse sensing mechanisms and provides a rigorous basis for comparing MS designs across materials and spectral regimes. Future progress will be driven by approaches that explicitly address physical limits rather than incremental resonance sharpening. Advances in low-loss and polaritonic materials, hybrid metal dielectric architectures, and actively tunable MSs will expand accessible parameter space for balancing confinement, quality factor, and robustness. At the same time, chemical selectivity is expected to increasingly rely on multimodal sensing, spectral and spatial multiplexing, and physics-informed data analysis rather than on intrinsic MS response alone.

Chemical selectivity in flatland MS gas sensing can be improved through multiplexed sensing strategies rather than relying on a single functional response. Sensor arrays incorporating different functional coatings enable pattern-based gas identification but introduce calibration complexity and cross-sensitivity to environmental variables such as humidity and temperature. Spectral multiplexing, where multiple resonances or spectral bands are monitored simultaneously, improves molecular discrimination but requires more complex optical design and readout instrumentation. Data-driven analysis, including machine learning, can further enhance selectivity by extracting correlated features from multidimensional sensor outputs, although performance depends strongly on training dataset quality and long-term sensor stability. Practical implementation will require balancing selectivity gains against fabrication complexity, system cost, and calibration requirements.

A prioritized research roadmap for the next 3–5 years can be defined across four primary challenges. First, materials and optical loss remain fundamental performance limits. Progress is expected from low-loss polaritonic materials, protected plasmonic layers, and hybrid metal-dielectric resonator architectures that maintain strong field confinement while improving environmental and thermal stability. Second, scalable fabrication and system-level integration represent key translational barriers. Future efforts should prioritize nanoimprint and deep-UV lithography, wafer-scale pattern transfer, and CMOS-compatible fabrication workflows. Integration of MS structures with on-chip photodetectors and readout electronics will be critical for reducing system footprint and cost. Third, achieving robust chemical selectivity under realistic environmental conditions remains a key system-level challenge. Advances will likely rely on hybrid approaches combining functional surface chemistry, multimodal optical sensing, and data-driven spectral analysis. Coupled resonance shift and spectroscopic fingerprint detection is expected to improve molecular discrimination in complex gas environments. Fourth, cost and operational stability will determine large-scale deployment. Research priorities include robust packaging, low-power optical interrogation, and calibration-stable sensor architectures. Development of standardized testing and benchmarking protocols will be essential for cross-platform performance validation.

Equally important is the transition from component-level demonstrations to system-aware design. Co-optimization of the optical response, thermal stability, fabrication tolerance, and readout strategy will be essential for translating flatland MS sensors into deployable technologies. Integration with CMOS-compatible detectors, microelectromechanical platforms, and computational inference frameworks is likely to define the next generation of compact and scalable gas sensing systems. In this broader context, flatland MSs should be viewed not simply as nanophotonic structures, but as programmable optical interfaces that link gaseous environments to measurable optical states within planar platforms. Continued convergence of materials science, resonant photonics, and system-level engineering is expected to establish MS-enabled gas sensing as a foundational technology for future environmental, industrial, and healthcare monitoring applications.

## Figures and Tables

**Table 1:** Physical transduction mechanisms and performance trade-offs in flatland MS gas sensors.

Sensing Mechanism	Primary Physical Perturbation	MS Resonance Type	Dominant Optical Observable	Key Design Parameters	Spectral Regime	Strengths	Limitations/Trade-offs	Representative Gas Sensing Performance Indicators
Resonantly enhanced molecular absorption [42,69]	Gas-induced absorption loss (Im{ε}, κ)	Plasmonic or hybrid perfect absorber modes	Intensity attenuation/emissivity change	Mode overlap with gas, resonance linewidth, field enhancement factor	Mid-IR, THz	High chemical selectivity via vibrational fingerprints; ultracompact footprint	Material loss and thermal background; requires spectral alignment	Typically ppm to sub-ppm detection when aligned with molecular absorption bands; high selectivity; response time governed by gas diffusion and optical acquisition speed
Refractive index–induced resonance shift [19,75]	Δneff from gas density, adsorption, or infiltration	Mie resonances, guided-mode resonances, quasi-BIC modes	Spectral shift (Δλ or Δω)	Quality factor Q, mode volume Veff, field–gas overlap Γ	Visible–IR	Quantitative sensing; compatible with mature detector technologies	Thermal drift; high-Q modes sensitive to fabrication imperfections	Typically ppm detection limits; sub-ppm achievable in high-Q architectures; sensitivity commonly tens to hundreds nm/RIU; response depends on adsorption kinetics
Loss damping/linewidth modulation [76,77,78]	Additional non-radiative decay channels	Hybrid plasmonic resonances	Amplitude modulation/linewidth broadening	Radiative vs. non-radiative loss balance; intrinsic absorption	Visible–IR	Simple fixed-wavelength readout; fast temporal response	Poor intrinsic chemical specificity; baseline drift	ppm to % concentration detection depending on baseline stability; fast response possible; often used for threshold detection applications
Material-mediated chemo-optical modulation [22,71,79]	Gas-induced change in ε, birefringence, or conductivity of functional layer	Dielectric or hybrid MSs	Polarization change/intensity modulation	Functional layer thickness; diffusion kinetics; MS polarization sensitivity	Visible–IR	High sensitivity; tunable selectivity via functional material choice	Stability, aging, and response time limited by material chemistry	ppm to sub-ppm possible depending on functional layer; response/recovery dominated by diffusion and adsorption–desorption kinetics
Polarization-encoded wavefront modulation [80]	Gas-triggered polarization conversion	Anisotropic dielectric MSs	Holographic image switching/polarization state change	Jones matrix engineering; material birefringence control	Visible	Instrument-free visual readout; intuitive alarm systems	Limited quantitative capability; threshold-type response	Typically threshold or qualitative detection; suitable for safety or wearable alarm applications rather than quantitative sensing
Phase-transition-driven plasmonic switching [81]	Gas-induced metal–dielectric phase transition (e.g., Mg ↔ MgH_2_)	Localized and lattice plasmon modes	Reflectance or color change	Nanoparticle geometry; catalytic layer; diffusion length	Visible	Large visually observable signal; reversible switching	Material fatigue; gas-specific chemistry	ppm-level detection demonstrated for specific gases (e.g., H_2_); response time governed by phase-transition kinetics
Collective lattice resonance sensing [82,83]	Gas-induced perturbation of surface lattice resonances	Surface lattice resonances (SLRs)	Narrow spectral shift/extinction modulation	Array periodicity; particle size; dielectric environment	Visible–NIR	Narrow linewidth vs. isolated nanoparticles	Still limited by metallic ohmic loss	ppm detection achievable; improved spectral resolution vs. single-particle plasmon sensors
Hybrid metal–dielectric resonant sensing [20,22,69,75,84]	Environmental perturbation of dielectric-dominated modes	Hybrid Mie–plasmon resonances	Spectral shift with reduced loss	Energy distribution between metal and dielectric regions	Mid-IR	Higher Q with retained field confinement	Increased fabrication complexity	ppm to sub-ppm possible depending on Q-factor and functionalization strategy

**Table 2:** Optical material platforms for flatland MS gas sensing: electromagnetic response, polaritonic behavior, and intrinsic performance limits.

Material Platform	Representative Systems	Governing Electromagnetic Response	Supported Resonant/Polaritonic Modes	Dominant Spectral Regime	Functional Role in Flatland Gas Sensing	Fundamental Advantage	Intrinsic Physical Limitation
Noble metals [121,122,123]	Au, Ag, Al	Drude-like free-electron response with large Im{ε}	Localized surface plasmons, lattice plasmons	Visible–NIR	Extreme near-field confinement and absorption enhancement	Very high local field intensity	Ohmic loss fundamentally limits Q and SNR
Heavily doped semiconductors [124,125]	InAs, InSb, ITO	Tunable plasma frequency with reduced damping	Plasmon-like resonances	Mid-IR–THz	Spectral alignment with molecular vibrational bands	Lower loss than noble metals	Strong temperature dependence of carrier density
High-index dielectrics [27,126]	Si, Si_3_N_4_, TiO_2_	Low-loss displacement current response	Mie resonances, guided-mode resonances	Visible–IR	Resonance-shift-based refractive index sensing	High-Q, thermal stability	Limited intrinsic field enhancement
Polar dielectrics [127,128]	SiC, hBN	Strong optical phonon resonances (Reststrahlen band)	Surface phonon polaritons	Mid-IR	Low-loss confinement at molecular fingerprint frequencies	Orders-of-magnitude lower loss than metals	Narrow operational bandwidth
Hybrid metal–dielectric systems [116,129,130]	Au–Si, Al–Si_3_N_4_	Energy redistribution between lossy and low-loss media	Hybrid plasmon–Mie modes, quasi-BICs	IR	Optimized trade-off between confinement and Q	Enhanced sensitivity with reduced loss	Increased fabrication and design complexity
Two-dimensional materials [131,132,133,134]	Graphene, TMDs	Tunable surface conductivity (σ(ω))	Graphene plasmons, exciton–polaritons	THz–Mid-IR	Electrically tunable gas–plasmon coupling	Extreme field confinement and tunability	Environmental sensitivity and stability
Functional material overlays [44,135]	Liquid crystals, MOFs, metal oxides	Gas-induced change in ε, birefringence, or conductivity	Indirectly coupled MS resonances	Visible–IR	Chemical amplification and selectivity layer	High sensitivity and programmability	Diffusion-limited response and aging

## Data Availability

No new data were created or analyzed in this study.
